# Effects of Dietary Standardized Ileal Digestible Lysine and Amylose/Amylopectin Ratio on Intestinal Morphology, Barrier-Related Gene Expression, and Cecal Microbiota in Broilers Fed Low-Protein Diets

**DOI:** 10.3390/ani16121914

**Published:** 2026-06-20

**Authors:** Minhao Zhang, Jianmin Yuan

**Affiliations:** State Key Laboratory of Animal Nutrition and Feeding, College of Animal Science and Technology, China Agricultural University, Beijing 100193, China

**Keywords:** broiler, lysine, amylose/amylopectin ratio, intestinal health, low-protein diet

## Abstract

This study investigated the effects of dietary standardized ileal digestible (SID) lysine levels and amylose to amylopectin ratios on the intestinal health of broilers fed an 18.5% crude protein diet from 22 to 42 days of age. A total of 540 healthy male Ross 308 broilers were randomly assigned to nine treatments in a 3 × 3 factorial design consisting of three SID lysine levels (1.00%, 1.20%, and 1.40%) and three AM/AP ratios (0.19, 0.29, and 0.41), with six replicates of 10 birds each. Ileal morphology, intestinal barrier function and inflammation-related gene expression, and the composition of cecal microbiota were evaluated. Significant interactions between lysine level and AM/AP ratio were observed for Occludin, ZO-1, Claudin-1, and TNF-α expression, with the highest expression in the 1.40% lysine + 0.41 AM/AP group and the lowest in the 1.00% lysine + 0.19 AM/AP group. The VH/CD ratio showed a significant interaction, with the highest value in the 1.20% lysine + 0.19 AM/AP group and the lowest in the 1.40% lysine + 0.41 AM/AP group. IL-18 and IL-10 were primarily affected by the main effects of lysine and AM/AP ratio. The expression levels of both IL-10 and IL-18 increased with increasing lysine level and increasing starch AM/AP ratio. Dietary SID lysine level and AM/AP ratio interactively regulate the expression of barrier-related genes, inflammatory status, intestinal morphology, and cecal microbiota, potentially contributing to enhanced intestinal health in broilers. However, because microbial metabolites were not measured, the functional significance of the observed microbiota alterations remains speculative. In broilers fed an 18.5% CP diet, a combination of 1.20% SID lysine with an AM/AP ratio of 0.19 was identified as the optimal strategy for maintaining intestinal morphology from 22 to 42 days of age.

## Simple Summary

Reducing dietary protein levels is an effective strategy to improve the sustainability of broiler production, but it may also affect intestinal health. Lysine is an essential amino acid in broilers, commonly used as a reference standard amino acid in broilers, and the amylose/amylopectin (AM/AP) ratio, which determines starch digestion rate, may interact to modulate intestinal barrier function, inflammatory responses, and cecal microbiota. This study investigated the combined effects of dietary lysine level and starch composition on intestinal health in broilers fed an 18.5% crude protein diet. The results showed that lysine level and starch type interacted to influence intestinal morphology, barrier-related gene expression, and cecal microbial composition. In particular, a moderate lysine level combined with a low AM/AP ratio supported better intestinal structure. These findings provide useful information for optimizing low-protein diets and maintaining intestinal health in broiler chickens.

## 1. Introduction

With the decline in soybean imports in China, the development and promotion of low-protein diets have become a strategic priority in poultry nutrition. Because broilers have a high protein requirement, reducing dietary soybean meal while supplementing crystalline amino acids (AA) is a key approach to maintaining intestinal health and ensuring national feed security [1,2].

Lysine, an essential amino acid commonly used as a reference standard, not only participates in protein synthesis but also serves as an essential substrate for intestinal epithelial cell proliferation, tight junction protein expression, and immune function [3,4]. Dietary lysine deficiency impairs both cellular and humoral immunity, increases intestinal permeability, downregulates tight junction proteins, reduces the abundance of beneficial bacteria, and alters gut microbiota composition [5]. Supplementing lysine in low-protein diets significantly improves feed conversion ratio, restores growth and carcass performance to near-normal levels, and upregulates intestinal barrier gene expression [6,7,8,9,10]. However, excessive lysine reduces average daily gain and feed intake, leading to growth depression [11].

Starch is the primary energy source in broiler diets. A higher amylose/amylopectin (AM/AP) ratio slows starch digestion rate, allowing more starch to reach the hindgut for fermentation [12]. In particular, diets with higher amylose content are generally associated with increased resistant starch formation, which serves as an important substrate for cecal microbiota [13]. Resistant starch can selectively stimulate the growth of beneficial bacterial populations, including *Lactobacillus* and other saccharolytic microorganisms, thereby influencing microbial community structure and metabolic activity [14]. The fermentation of resistant starch by gut microbiota produces short-chain fatty acids (SCFAs), such as acetate, propionate, and butyrate, which contribute to intestinal epithelial energy supply, maintenance of barrier integrity, and modulation of immune responses [15,16]. Consequently, starch digestive characteristics may indirectly affect intestinal barrier gene expression, inflammatory balance, and microbial composition. Furthermore, previous studies suggested that a high AM/AP ratio enables slower glucose release, which better synchronizes with amino acid absorption, thereby reducing AA catabolism in the intestinal mucosa and promoting protein synthesis [17].

Although existing studies have investigated not only the individual effects of dietary lysine levels and AM/AP ratios [6,12,16], but also the interactions between amino acid nutrition and carbohydrate digestion kinetics on nutrient utilization and growth performance in broilers [9,10], research focusing on their combined effects on intestinal health remains limited. In particular, little information is available regarding how dietary SID lysine and AM/AP ratio interact to influence intestinal morphology, barrier function, immune responses, and cecal microbial communities in broilers fed low-protein diets. Therefore, further investigation is needed to elucidate the potential synergistic effects of amino acid and carbohydrate nutritional strategies on intestinal health.

This study employed a 3 × 3 factorial design to investigate the effects of different AM/AP ratios (0.19, 0.29, 0.41) and standardized ileal digestible (SID) lysine levels (1.00%, 1.20%, 1.40%) on: ileal tight junction protein gene expression (Occludin, ZO-1, Claudin-1), inflammatory cytokine genes (IL-18, TNF-α, IL-10), ileal morphology, and cecal microbiota composition in broilers. The aim is to provide theoretical support for optimizing lysine and starch composition in low-protein broiler diets to enhance intestinal health.

## 2. Materials and Methods

### 2.1. Animal Ethics Statement

All animal procedures were performed at the Zhuozhou Poultry Nutrition Research Base of China Agricultural University (Hebei, China). The experimental protocols were approved by the Laboratory Animal Ethical Committee of China Agricultural University (No: AW90504202-1-2, approved on 9 May 2024) and conducted in accordance with the Beijing Regulations of Laboratory Animals.

### 2.2. Experimental Design and Diet Formulation

A total of 540 healthy male Ross 308 broiler chicks (1 d of age) were fed a common starter diet until 21 d of age. At 21 d of age, birds were balanced by body weight (average 0.951 ± 0.003 kg) and randomly allotted to 9 treatment groups in a 3 × 3 factorial arrangement. The treatments consisted of 3 different AM/AP ratios (0.19, 0.29, and 0.41 (provided by waxy corn, corn, and pea starch, respectively) with 3 different SID lysine levels (1.00%, 1.20%, and 1.40%). Each treatment had 6 replicates with 10 birds per replicate. All diets were formulated to meet or exceed Ross 308 nutrient requirements (22–42 d). The basal SID lysine level was set at 1.20% based on preliminary data, with ±0.20% fluctuations. Other essential amino acids were balanced according to the ideal AA profile. Prior to diet formulation, feed ingredients were analyzed for nutrient composition using near-infrared reflectance spectroscopy (NIRS) by Evonik Operations GmbH (Beijing, China). Ingredient composition and nutrient levels are shown in Table 1.

### 2.3. Bird Management

Management followed the Ross 308 Broiler Management Handbook. The ambient temperature was gradually decreased from 33 °C to 20 °C over 42 d. Photoperiod was 23L:1D (d 1–3 and d 32–42) and 20L:4D (d 4–31). Broilers had ad libitum access to feed and water.

### 2.4. Sample Collection

At 42 d of age, one bird per replicate with an average body weight was selected. Birds were electrically stunned and exsanguinated. Middle ileal segments (~3 cm) were collected, rinsed with ice-cold PBS, and stored at −80 °C for gene expression and morphology. Ileal digesta were also collected. Cecal digesta were snap-frozen in liquid nitrogen and stored at −80 °C for 16S rRNA gene sequencing.

### 2.5. Intestinal Morphology

Ileal segments were fixed in 4% paraformaldehyde, paraffin-embedded, sectioned at 5 μm, and stained with hematoxylin and eosin (H&E). Villus height (VH) and crypt depth (CD) were measured using an optical microscope and Image-Pro Plus software 7.0 (Media Cybernetics, Inc., Rockville, MD, USA). The villus height to crypt depth ratio (VH/CD) was calculated. Ten well-oriented villi and crypts per section were measured.

### 2.6. Quantitative Real-Time PCR (qRT-PCR)

Total RNA was extracted from ileal tissue using TRIzol reagent (Promaga company, Shanghai, China). cDNA was synthesized, and qRT-PCR was performed using SYBR Green II on an Applied Biosystems 7300 system (Waltham, MA, USA). Primer sequences were designed based on published chicken gene sequences obtained from GenBank and are listed in Table 2. Primer specificity was confirmed by the presence of a single amplification product. Melt-curve analysis was performed at the end of each qPCR run, and all reactions exhibited a single sharp peak, indicating specific amplification. Relative mRNA expression was normalized to β-actin and calculated using the 2^−ΔΔCt^ method.

### 2.7. 16S rRNA Gene Sequencing

For microbiota analysis, four representative treatment groups were selected based on preliminary growth performance results obtained under the same experimental conditions (unpublished data). These groups comprised combinations of two SID lysine levels (1.20% and 1.40%) and two amylose/amylopectin (AM/AP) ratios (0.19 and 0.41). One bird from each replicate was sampled at 42 d of age, resulting in six biological replicates per treatment (*n* = 6). Therefore, a total of 24 cecal digesta samples were subjected to microbiota analysis.

Microbial DNA was extracted from cecal digesta using a commercial kit. The V3–V4 region of the 16S rRNA gene was amplified. PCR products were purified and sequenced on the Illumina MiSeq platform (San Diego, CA, USA). Raw sequencing reads were processed using QIIME2 (version 2019.4). Primer sequences were removed using the q2-cutadapt plugin, and reads without correctly matched primer sequences were discarded. Sequence filtering criteria included removal of low-quality reads, reads containing ambiguous bases (N), chimeric sequences, and sequences that failed the DADA2 quality-control procedure. Subsequently, denoising, paired-end merging, and chimera removal were performed using the DADA2 plugin to generate amplicon sequence variants (ASVs). Singleton ASVs were removed prior to downstream analyses. Taxonomic assignment was performed against the SILVA reference database using the QIIME2 classify-sklearn classifier with a confidence threshold of 0.7. Alpha and beta diversity analyses were performed. Taxonomic annotation used the Silva database.

The sequencing data have been deposited in the NCBI Sequence Read Archive (SRA) under accession number PRJNA1475828.

### 2.8. Statistical Analysis

Data were analyzed using IBM SPSS Statistics 26.0. Prior to statistical analysis, data were assessed for normality using the Shapiro–Wilk test and for homogeneity of variances using Levene’s test. A 3 × 3 factorial arrangement was analyzed by two-way ANOVA using the General Linear Model (GLM), including main effects of AM/AP ratio, SID lysine level, and their interaction. For intestinal morphology, gene expression, and cecal microbiota analyses, one bird was randomly selected from each replicate pen at the end of the experiment. Thus, each sampled bird represented a different replicate pen, and six observations per treatment were used in the statistical analyses. When a significant interaction was observed (*p* < 0.05), simple effects were analyzed using Duncan’s multiple range test. Significance was declared at (*p* < 0.05), and (0.05< *p* < 0.10) was considered a trend.

## 3. Results

### 3.1. Ileal Barrier and Inflammatory Factor Gene Expression

This study revealed significant interactions between dietary SID lysine level and AM/AP ratio on the mRNA expression of Occludin, ZO-1, and Claudin-1 (*p* < 0.05) (Table 3). The highest expression of all three tight junction genes was observed in the 1.40% lysine + 0.41 AM/AP group, and the lowest in the 1.00% lysine + 0.19 AM/AP group.

For TNF-α, a significant interaction was also observed (*p* < 0.05), with the highest expression in the 1.40% lysine + 0.41 AM/AP group and the lowest in the 1.00% lysine + 0.19 AM/AP group. IL-18 and IL-10 were primarily affected by the main effects of lysine level (*p* < 0.001) and AM/AP ratio (*p* < 0.05), with higher lysine and higher AM/AP increasing expression.

### 3.2. Ileal Morphology

A significant interaction between lysine level and AM/AP ratio was observed for the VH/CD ratio (*p* < 0.05) (Table 4). The highest VH/CD was in the 1.20% lysine + 0.19 AM/AP group, and the lowest was in the 1.40% lysine + 0.41 AM/AP group. VH was significantly affected by lysine level (*p* = 0.003) and AM/AP ratio (*p* = 0.043), with the highest VH at 1.20% lysine and 0.19 AM/AP. CD was not significantly affected by any factor (*p* > 0.05).

### 3.3. Cecal Microbiota

**Figure 1:**
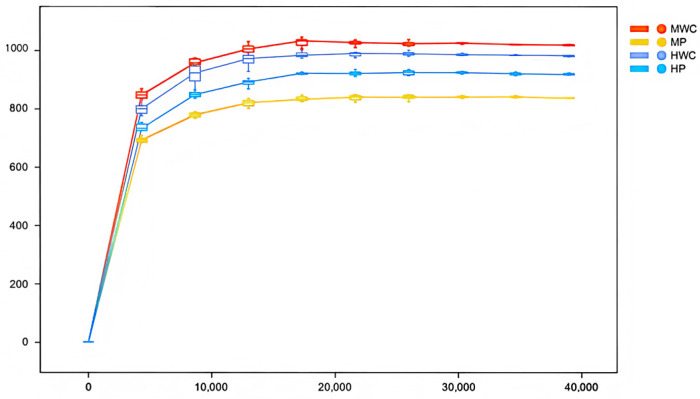
Rarefaction curves of cecal microbiota.

**Figure 2:**
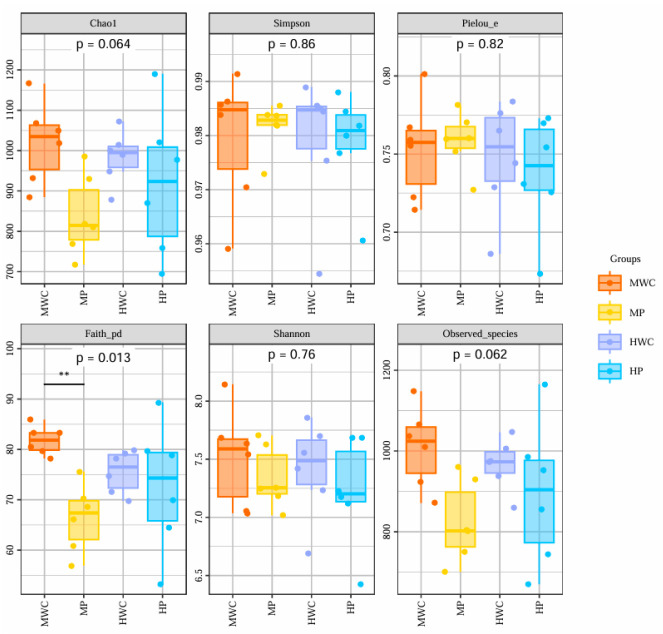
Alpha diversity of cecal microbiota. ** *p* < 0.01.

**Figure 3:**
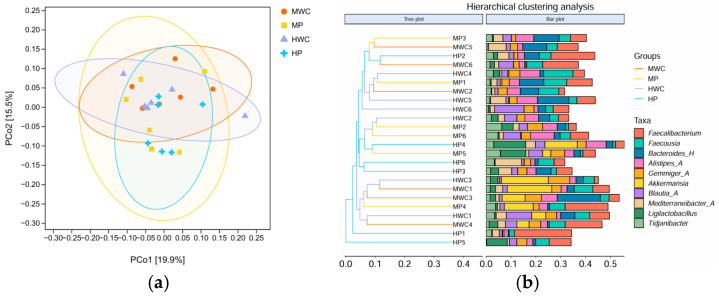
Beta Diversity of Gut Microbiota Across Different Starch Types and SID Lys Levels. (**a**) PCoA Analysis of Gut Microbiota. (**b**) Hierarchical Cluster Analysis of Gut Microbiota.

**Figure 4:**
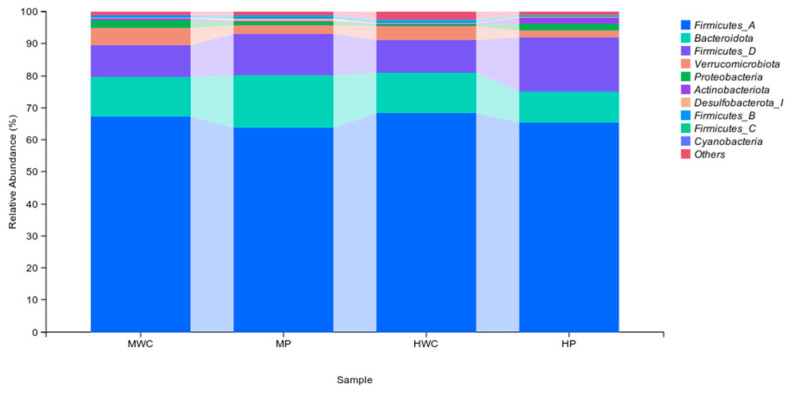
Phylum-level analysis of cecal microbial community structure.

**Figure 5:**
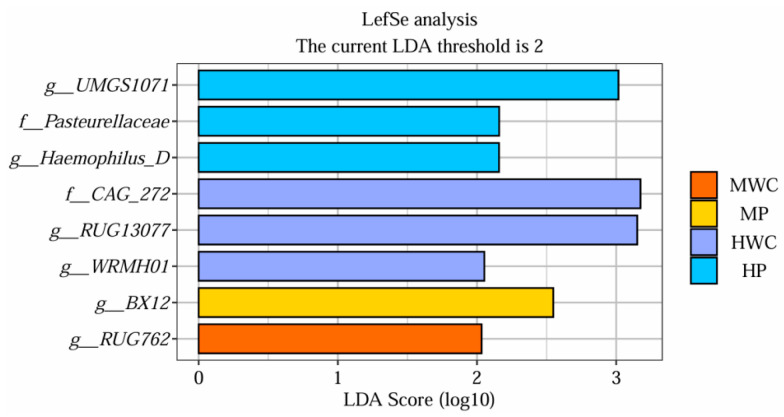
LEfSe analysis of cecal microbiota.

Sequencing of the V3–V4 region of the 16S rRNA gene yielded high-quality sequences (coverage > 99.8%) (Figure 1). High-throughput sequencing of the V3–V4 region of the 16S rRNA gene was performed on cecal digesta samples from the MWC (waxy corn + 1.20% SID lysine), MP (pea starch + 1.20% SID lysine), HWC (waxy corn + 1.40% SID lysine), and HP (pea starch + 1.40% SID lysine) groups. A total of 350,819 raw sequences were obtained. After quality filtering, denoising, sequence merging, and chimera removal, 325,255 valid sequences were retained, corresponding to an overall effective sequence rate of 92.71%. The numbers of raw sequences obtained from the MWC, MP, HWC, and HP groups were 83,630, 82,557, 88,898, and 95,734, respectively, while the corresponding numbers of valid sequences were 77,286, 76,906, 82,219, and 88,844 (Table 5). The effective sequence rates ranged from 92.41% to 93.16% across all groups (Table 5), indicating high sequencing quality and sufficient sequencing depth for subsequent analyses of microbial community composition and diversity. Alpha diversity analysis showed that the MWC group had significantly higher Faith’s PD index than the MP group (*p* = 0.013), with trends for higher Chao1 and observed species (*p* = 0.064 and 0.062, respectively) (Figure 2). Beta diversity (PCoA and ANOSIM) showed significant separation between MWC and MP (*p* = 0.021), MWC and HWC (*p* = 0.034), MWC and HP (*p* = 0.021), and HWC and HP (*p* = 0.036) (Figure 3) (Table 6). At the phylum level, *Firmicutes*, *Bacteroidota*, *Verrucomicrobiota*, and *Proteobacteria* dominated (>95% relative abundance) (Figure 4). LEfSe analysis further identified key discriminative taxa among the four groups (Figure 5).

## 4. Discussion

The ileum is a key site for host-microbe interactions, and its physical, chemical, and microbial barriers are critical for intestinal health [18]. Tight junction proteins (Occludin, ZO-1, Claudin-1) maintain epithelial integrity; their increased expression generally reflects reduced permeability and enhanced barrier function [19]. Lysine is essential for intestinal epithelial cell proliferation and post-translational modification of tight junction proteins [4,6]. Starch with a higher AM/AP ratio resists rapid digestion, prolonging hindgut fermentation and increasing SCFA production, which can upregulate barrier genes [15]. In this study, the significant interactions between lysine and AM/AP ratio on Occludin, ZO-1, and Claudin-1 confirm that both factors synergistically regulate barrier function. The highest expression was in the 1.40% lysine + 0.41 AM/AP group, suggesting that higher lysine supply combined with a higher AM/AP ratio may stimulate the transcription of barrier-related genes. This aligns with Jiang et al. [20] in mice and Gao et al. [21] in piglets. This molecular response was not accompanied by improvements in intestinal morphology. It should be noted that tight-junction markers were evaluated only at the mRNA level in the present study, and transcriptional changes do not necessarily reflect protein abundance or functional barrier integrity. However, Yang et al. [22] found no effect of AM/AP ratio on barrier genes, possibly due to insufficient dose.

TNF-α is a pro-inflammatory cytokine, whereas IL-10 is generally regarded as an anti-inflammatory cytokine, and IL-18 participates in the regulation of immune responses through activation of Th1-associated pathways [23]. The significant interaction observed in TNF-α expression suggests that the dietary SID lysine level and the AM/AP ratio jointly influenced intestinal immune status. Because elevated TNF-α is commonly associated with inflammatory activation, the increased expression observed in the high lysine and high AM/AP treatment may indicate a degree of immune stimulation. However, the increased TNF-α at high lysine + high AM/AP differs from Mine et al. [24], possibly due to lysine-arginine antagonism at excess lysine levels [25]. The main effects of lysine and AM/AP on IL-18 and IL-10 align with Han [26] and Fan [27], supporting that these dietary factors may contribute to the regulation of intestinal immune homeostasis. However, the precise mechanisms underlying these responses remain to be further elucidated.

Ileal VH and VH/CD are positively correlated with nutrient absorption capacity [28,29]. The significant interaction on VH/CD, with the highest value at 1.20% lysine + 0.19 AM/AP, indicates that moderate lysine and rapid-fermenting starch optimize morphology. High lysine (1.40%) combined with high AM/AP (0.41) reduced VH/CD, possibly due to altered energy partitioning or excessive hindgut fermentation [30]. This is partially consistent with Gao et al. [21] but contrasts with studies showing high AM/AP improves morphology, suggesting species-specific and dose-dependent effects. Interestingly, the dietary treatment that produced the highest tight-junction gene expression was not the same as that yielding the most favorable intestinal morphology. Moreover, the treatment exhibiting the highest TNF-α expression also showed the lowest VH/CD ratio, suggesting that enhanced expression of certain barrier-related genes may not necessarily translate into improved intestinal health when accompanied by increased pro-inflammatory signaling. Tight junction-related gene expression represents a transcriptional response at the cellular level and may reflect adaptive or compensatory mechanisms triggered by dietary stimuli. Therefore, increased expression of Occludin, ZO-1, and Claudin-1 does not necessarily indicate improved intestinal function. In contrast, villus height and VH/CD ratio reflect structural adaptations of the intestinal mucosa and are more directly associated with absorptive surface area and nutrient utilization efficiency. The observation that the high lysine (1.40%) and high AM/AP (0.41) treatment induced the highest expression of barrier-related genes but the lowest VH/CD ratio suggests that transcriptional and morphological responses may not always occur in parallel. Given that villus height and VH/CD ratio are directly associated with absorptive surface area and nutrient utilization efficiency, intestinal morphology was considered a more biologically relevant parameter for evaluating overall intestinal function and formulating practical nutritional recommendations in the present study.

The cecal microbiota plays a pivotal role in broiler intestinal health. Previous studies have demonstrated that cecal microorganisms can ferment undigested carbohydrates into short-chain fatty acids (SCFAs), which have been reported to serve as the primary energy source for colonocytes, upregulate tight junction protein expression, modulate inflammatory responses, and ultimately enhance nutrient absorption and overall gut barrier [31]. Cecal microbiota composition was dominated by *Firmicutes, Bacteroidota*, *Verrucomicrobiota*, and *Proteobacteria*, similar to previous reports [32]. The significant differences in beta diversity between starch types (MWC vs. MP) indicate that starch source and AM/AP ratio alter microbial community structure. These microbial changes may potentially be associated with differences in microbial metabolic activity, intestinal barrier function, and inflammatory status [32]; however, the underlying mechanisms, including the possible involvement of SCFAs, require further investigation. Under low dietary SID lysine levels, the genera *g_RUG762* and *g_BX12* were enriched in the cecal microbiota. *G_RUG762* is reported to participate in protein and amino acid metabolism [33], while *g_BX12* has been negatively associated with pro-inflammatory markers [34], suggesting these taxa may be linked to intestinal physiological status. A limitation of the present study was that cecal SCFA concentrations were not measured. Therefore, although changes in microbial composition were observed, the functional consequences of these alterations on microbial metabolite production could not be directly evaluated. Future studies integrating microbiome analysis with SCFA quantification and metabolomic approaches would help clarify the mechanisms linking dietary SID lysine levels, the AM/AP ratio, the gut microbiota, and intestinal health.

Furthermore, it should be noted that manipulating the dietary SID lysine level and AM/AP ratio required adjustments to ingredient composition to maintain similar dietary AME, CP, and amino acid balance. Consequently, variations in starch sources may have altered starch digestibility characteristics and resistant starch content, while minor differences in dietary fiber and non-starch polysaccharides may also have influenced intestinal morphology and microbial composition. Therefore, the observed responses should be interpreted as the combined outcome of practical dietary strategies used to achieve the target SID lysine levels and AM/AP ratios, rather than as effects entirely independent of ingredient composition.

## 5. Conclusions

In broilers fed an 18.5% CP low-protein diet, dietary SID lysine level and AM/AP ratio interact to regulate ileal barrier gene expression, inflammatory cytokines, and ileal morphology.

High lysine (1.40%) combined with a high AM/AP ratio (0.41) maximizes tight junction (Occludin, ZO-1, Claudin-1) and TNF-α expression but reduces VH/CD ratio. Moderate lysine (1.20%) with a low AM/AP ratio (0.19) optimizes ileal villus height and VH/CD.

Under the conditions of the present study, in which broilers were fed an 18.5% CP low-protein diet from 22 to 42 days of age, a combination of 1.20% SID lysine and an AM/AP ratio of 0.19 is recommended to support intestinal morphology, while 1.40% lysine with 0.41 AM/AP may enhance barrier gene expression at the cost of morphological integrity.

## Figures and Tables

**Table 1:** Ingredient composition and nutrient levels of the experimental diets for broilers from 22 to 42 d of age (%, as-fed basis).

SID Lys level ^1^	1.00			1.20			1.40		
AM/AP ratio ^2^	0.19	0.29	0.41	0.19	0.29	0.41	0.19	0.29	0.41
Corn, AM/AP ratio = 0.29 ^2^	33.72	52.20	42.70	43.36	52.60	43.10	34.50	53.00	43.40
Soybean meal	18.75	19.56	10.90	17.03	17.44	8.80	14.55	15.34	6.69
Soybean oil	3.77	4.47	2.66	4.56	4.91	3.09	4.64	5.34	3.60
Corn gluten meal	5.00	5.00	5.00	5.00	5.00	5.00	5.00	5.00	5.00
Dicalcium phosphate	1.16	1.21	1.09	1.21	1.23	1.11	1.21	1.25	1.13
Limestone	1.01	0.98	1.07	1.00	0.99	1.08	1.03	1.00	1.10
Sodium chloride	0.30	0.30	0.30	0.30	0.30	0.30	0.30	0.30	0.30
Vitamins premix ^3^	0.03	0.03	0.03	0.03	0.03	0.03	0.03	0.03	0.03
Mineral premix ^4^	0.20	0.20	0.20	0.20	0.20	0.20	0.20	0.20	0.20
Choline chloride (50%)	0.15	0.15	0.15	0.15	0.15	0.15	0.15	0.15	0.15
Phytase 10,000, U/g	0.03	0.03	0.03	0.03	0.03	0.03	0.03	0.03	0.03
Antioxidant	0.02	0.02	0.02	0.02	0.02	0.02	0.02	0.02	0.02
*L*-Lysine hydrochloride (98%)	0.42	0.40	0.35	0.73	0.72	0.67	1.06	1.04	0.99
*DL*-Methionine (98%)	0.24	0.24	0.28	0.36	0.36	0.40	0.47	0.48	0.51
*L*-Threonine (98%)	0.09	0.09	0.11	0.25	0.25	0.27	0.41	0.41	0.44
*L*-Valine (98%)	-	0.01	0.05	0.20	0.20	0.24	0.39	0.40	0.45
*L*-Isoleucine (98%)	-	0.01	0.05	0.19	0.19	0.23	0.35	0.36	0.40
*L*-Arginine (98%)	0.11	0.10	0.01	0.38	0.38	0.28	0.66	0.65	0.56
Flour	15.00	15.00	15.00	15.00	15.00	15.00	15.00	15.00	15.00
Pea, AM/AP ratio = 0.76 ^2^	0.00	0.00	20.00	0.00	0.00	20.00	0.00	0.00	20.00
Waxy corn, AM/AP ratio = 0.05 ^2^	20.00	0.00	0.00	10.00	0.00	0.00	20.00	0.00	0.00
Total	100	100	100	100	100	100	100	100	100
Nutritional levels ^1^									
AME, Mcal/kg	3.20	3.20	3.20	3.20	3.20	3.20	3.20	3.20	3.20
CP, %	18.50	18.50	18.50	18.50	18.50	18.50	18.50	18.50	18.50
Calcium, %	0.70	0.70	0.70	0.70	0.70	0.70	0.70	0.70	0.70
NPP, %	0.30	0.30	0.30	0.30	0.30	0.30	0.30	0.30	0.30
SID Lys, %	1.00	1.00	1.00	1.20	1.20	1.20	1.40	1.40	1.40
SID Met, %	0.52	0.52	0.52	0.63	0.63	0.63	0.73	0.73	0.73
SID Met + Cys, %	0.79	0.79	0.78	0.89	0.89	0.87	0.98	0.98	0.96
SID Thr, %	0.64	0.64	0.64	0.77	0.77	0.77	0.90	0.90	0.90
SID Val, %	0.75	0.75	0.75	0.90	0.90	0.90	1.06	1.06	1.06
SID Ile, %	0.67	0.67	0.67	0.81	0.81	0.81	0.94	0.94	0.94
SID Arg, %	1.04	1.04	1.04	1.25	1.25	1.25	1.46	1.46	1.46
Total starch, % ^2^	45.57	40.71	40.52	45.83	37.03	35.74	39.24	37.52	37.12

AME, apparent metabolizable energy; CP, crude protein; NPP, non-phytate phosphorus; SID, standardized ileal digestibility. ^1^ Nutrient levels were calculated based on the ingredient compositions analyzed by near-infrared reflectance spectroscopy. ^2^ Actually measured values of nutrient components. ^3^ The vitamin premix provided (per kg of diets) the following: vitamin A, 15,000 IU, vitamin D3, 3600 IU; vitamin E, 30 IU; vitamin K3, 3.00 mg; vitamin B2, 9.60 mg; vitamin B12, 0.03 mg; biotin, 0.15 mg; folic acid, 1.50 mg; pantothenic acid, 13.80 mg; nicotinic acid, 45 mg. ^4^ The trace mineral premix provided (per kg of diets) the following: Cu, 16 mg; Zn, 110 mg; Fe, 80 mg; Mn, 120 mg; Se, 0.30 mg; I, 1.50 mg.

**Table 2:** Primer sequences for qRT-PCR.

Gene	GenBank Accession	Forward Sequences (5′–3′)	Reverse Sequences (5′–3′)
*β*-actin	NM_205518.2	CCACCGCAAATGCTTCTAAAC	AAGACTGCTGCTGACACCTTC
*ZO-1*	NM_001301025.3	CTTCAGGTGTTTCTCTTCCTCCTC	CTGTGGTTTCATGGCTGGATC
*Occludin*	NM_205128.1	ACGGCAGCACCTACCTCAA	GGGCGAAGAAGCAGATGAG
*Claudin-1*	NM_001013611.2	TACAGCCCTTGGCCAATACA	CCAAGAAACAACCACCAGCA
IL-18	NM_204608.3	GTGTGTGCAGTACGGCTTAG	TCCACTGCCAGATTTCACCT
TNF-α	NM_204267.2	CCCCTACCCTGTCCCACAA	TGAGTACTGCGGAGGGTTCAT
IL-10	NM_001004414.4	CAGACCAGCACCAGTCATCAG	ATCCCGTTCTCATCCATCTTCTCG

**Table 3:** Ileal barrier and inflammatory factor mRNA expression (means ± SEM).

Item		Occludin	ZO-1	Claudin-1	IL-18	TNF-α	IL-10
1.00% SID Lys	AM/AP: 0.19	0.162 ^c^	0.267 ^e^	0.119 ^d^	0.168	0.141 ^b^	0.329
	AM/AP: 0.29	0.186 ^c^	0.157 ^e^	0.246 ^cd^	0.168	0.179 ^b^	0.268
	AM/AP: 0.41	0.868 ^b^	1.541 ^cd^	2.139 ^a^	1.247	0.952 ^a^	2.662
1.20% SID Lys	AM/AP: 0.19	1.184 ^a^	1.898 ^bc^	0.983 ^bc^	1.197	0.960 ^a^	1.229
	AM/AP: 0.29	1.026 ^ab^	1.081 ^d^	1.081 ^b^	1.034	0.893 ^a^	1.468
	AM/AP: 0.41	0.757 ^b^	1.586 ^c d^	0.951 ^bc^	1.520	1.060 ^a^	2.661
1.40% SID Lys	AM/AP: 0.19	0.921 ^ab^	1.884 ^b c^	1.415 ^b^	1.618	0.844 ^a^	2.963
	AM/AP: 0.29	0.956 ^ab^	2.421 ^b^	0.786 ^bcd^	1.511	1.095 ^a^	2.049
	AM/AP: 0.41	1.189 ^a^	3.505 ^a^	1.966 ^a^	2.147	1.103 ^a^	4.791
SEM		0.058	0.150	0.117	0.097	0.059	0.258
SID Lys	1.00%	0.405	0.655	0.835	0.527 ^c^	0.844	1.086 ^b^
	1.20%	0.989	1.522	1.005	1.251 ^b^	1.095	1.786 ^b^
	1.40%	1.022	2.603	1.389	1.759 ^a^	1.103	3.268 ^a^
AM/AP	0.19	0.756	1.350	0.839	0.994 ^c^	0.424	1.507 ^b^
	0.29	0.723	1.220	0.704	0.904 ^c^	0.971	1.262 ^b^
	0.41	0.938	2.211	1.686	1.638 ^b^	1.014	3.371 ^a^
*p* Value							
SID Lys		<0.001	<0.001	0.14	<0.001	<0.001	0.001
AM/AP		0.273	0.011	<0.001	0.002	0.014	0.001
AM/AP × SID Lys		<0.001	0.001	<0.001	0.147	0.004	0.675

Different letters in shoulder markers indicate significant differences (*p* < 0.05), while the same letters indicate no significant difference (*p* > 0.05).

**Table 4:** Ileal Intestinal Morphology.

Item		Villus Height, μm	Crypt Depth, μm	VH/CD
1.00% SID Lys	AM/AP: 0.19	658.61	138.5	4.84 ^bc^
	AM/AP: 0.29	601.88	129.2	4.73 ^bc^
	AM/AP: 0.41	618.78	130.39	4.84 ^bc^
1.20% SID Lys	AM/AP: 0.19	785.52	131.76	6.04 ^a^
	AM/AP: 0.29	678.5	135.82	5.10 ^b^
	AM/AP: 0.41	619.16	139.34	4.69 ^bc^
1.40% SID Lys	AM/AP: 0.19	594.21	134.18	4.46 ^cd^
	AM/AP: 0.29	578.14	141.78	4.13 ^de^
	AM/AP: 0.41	550.46	144.2	3.91 ^e^
SID Lys	1.00%	626.42 ^b^	132.7	4.81 ^b^
	1.20%	694.40 ^a^	165.64	5.28 ^a^
	1.40%	574.27 ^b^	140.05	4.17 ^c^
AM/AP	0.19	679.45 ^a^	134.81	5.11 ^a^
	0.29	619.51 ^ab^	135.6	4.65 ^b^
	0.41	596.13 ^b^	137.98	4.48 ^b^
*p* value				
SID Lys		0.003	0.502	<0.001
AM/AP		0.043	0.871	<0.001
AM/AP × SID Lys		0.48	0.732	0.004

Different letters in shoulder markers indicate significant differences (*p* < 0.05), while the same letters indicate no significant difference (*p* > 0.05).

**Table 5:** Sequencing Data Quality Assessment.

Item	Raw Sequences	Valid Sequences	Effective Sequence Rates, %
MWC	83,630	77,286	92.41
MP	82,557	76,906	93.16
HWC	88,898	82,219	92.49
HP	95,734	88,844	92.80

**Table 6:** ANOSIM results for cecal microbiota beta diversity (unweighted unifrac).

Group1	Group2	R	*p*-Value
MWC	MP	0.207407	0.021
MWC	HWC	0.196296	0.034
MWC	HP	0.211111	0.021
MP	HWC	0.090741	0.088
MP	HP	0.090741	0.065
HWC	HP	0.133333	0.036

## Data Availability

The original contributions presented in this study are included in the article. Further inquiries can be directed to the corresponding author.

## Abbreviations

SID: Standardized Ileal Digestible; AM/AP: Amylose/Amylopectin ratio; Ross 308: Ross 308 broiler; NIRS: Near-infrared reflectance spectroscopy; VH: Villus Height; CD: Crypt Depth; VH/CD: Villus Height to Crypt Depth ratio; H&E: Hematoxylin and eosin; qRT-PCR: Quantitative real-time PCR; OTUs: Operational Taxonomic Units; PCoA: Principal Coordinate Analysis; ANOSIM: Analysis of Similarities; GLM: General Linear Model; ANOVA: Analysis of Variance; MWC: Waxy corn + 1.20% lysine; MP: Pea starch + 1.20% lysine; HWC: High waxy corn; HP: High pea starch; Faith’s PD: Faith’s Phylogenetic Diversity; IL-18: Interleukin-18; TNF-α: Tumor Necrosis Factor-α; IL-10: Interleukin-10; Occludin: Occludin; ZO-1: Zonula Occludens-1; Claudin-1: Claudin-1; SCFA: Short-chain fatty acid; mTORC1: mammalian target of rapamycin complex 1.
